# Impact of *TLR9* and *TLR7* gene polymorphisms on prognosis and survival of patients with oral squamous cell carcinoma

**DOI:** 10.17305/bb.2024.10550

**Published:** 2024-12-01

**Authors:** Miroslav Obrenović, Gordana Šupić, Satoru Miyabe, Irena Mladenović, Ružica Kozomara, Saša Jović, Aleksandra Petković Ćurčin, Debora Štefik, Srboljub Stosić, Biserka Vukomanović Ðurđević

**Affiliations:** 1 Faculty of Medicine, University of East Sarajevo, Foča, Bosnia and Herzegovina; 2 Department for ENT and Maxillofacial Surgery, University Hospital Foča, Foča, Bosnia and Herzegovina; 3 Medical Faculty of Military Medical Academy, University of Defence, Belgrade, Serbia; 4 Institute for Medical Research, Military Medical Academy, Belgrade, Serbia; 5 Department of Maxillofacial Surgery, School of Dentistry, Aichi Gakuin University, Nagoya, Japan; 6 Department of Prosthodontics, University of East Sarajevo, Foča, Bosnia and Herzegovina; 7 Clinic for Maxillofacial Surgery, Military Medical Academy, Belgrade, Serbia; 8 Institute for Pathology and Forensic Medicine, Military Medical Academy, Belgrade, Serbia

**Keywords:** *TLR9*, *TLR7*, polymorphisms, oral squamous cell carcinoma (OSCC), prognosis, survival, oral cancer risk

## Abstract

Despite significant efforts in developing new diagnostic and therapeutic modalities, oral squamous cell carcinomas (OSCCs) still exhibit a high recurrence rate, a low five-year survival rate, and an increasing prevalence. Toll-like receptors (TLRs), which initiate and perpetuate immune mechanisms upon activation, have been linked to immune surveillance and the antitumor immune response. The aim of this study was to investigate the association between the polymorphisms of the *TLR7* rs3853839 and *TLR9* rs187084 genes and OSCC risk, clinicopathological features, and survival. Genotyping was assessed by real-time polymerase chain reaction (PCR) in 95 HPV-negative OSCC patients and 107 age- and sex-matched healthy controls. Patients with lymph node metastases had higher frequencies of the *TLR9* rs187084 CC variant genotype compared to the major TT genotype (*P* ═ 0.020) and to T-allele carriers (combined TT + CT genotypes, *P* ═ 0.015). A higher prevalence of advanced stage III was observed in patients with the *TLR9* rs187084 variant CC genotype compared to TT (*P* ═ 0.047) and to T-allele carriers (TT + CT, *P* ═ 0.037). Kaplan–Meier analysis revealed a lower overall survival (OS) rate in patients with the *TLR9* rs187084 variant CC genotype compared to the TT genotype (*P* ═ 0.010, log-rank test) and to T-allele carriers (TT + CT genotypes, *P* ═ 0.002), though it was not an independent predictor of OS. Both *TLR9* rs187084 and *TLR7* rs3853839 polymorphisms were associated with high alcohol consumption (*P* ═ 0.027 and *P* ═ 0.001, respectively). The investigated genetic variations were not associated with OSCC susceptibility. The variant CC genotype of the *TLR9* rs187084 polymorphism might be a marker of poor survival and tumor progression in OSCC.

## Introduction

Oral squamous cell carcinoma (OSCC), a subgroup of head and neck squamous cell carcinoma (HNSCC), is the eighth most common cancer worldwide, characterized by a high recurrence rate, a low five-year survival rate, approximately 50%, and an increasing prevalence worldwide [1]. Several factors contribute to the development of oral cancer, including smoking, alcohol consumption and their synergistic effect, human papillomavirus (HPV) infection, chronic inflammation, and the accumulation of genetic and epigenetic changes [2–4]. The HPV-negative status of oropharyngeal carcinomas is associated with a significantly poor prognosis and poor response to treatment [5], which has led to the classification of oropharyngeal tumors according to their HPV status and the creation of a separate staging system [6, 7]. However, the significance of HPV status for the survival of OSCC patients is still controversial. While high-risk HPV positivity could be a good predictor of the five-year survival rate of OSCC patients [8, 9], another study found that HPV status had no impact on OSCC prognosis, except in patients with recurrent or progressive disease, where HPV positivity was significantly associated with improved survival compared to HPV-negative OSCC patients [10]. Emerging evidences confirm that genetic variations in immune-related signaling pathways in both tumor and immune cells play a key role in the immune landscape, and influence the antitumor immune response and tumor progression.

Toll-like receptors (TLRs) represent a family of transmembrane proteins involved in both innate and adaptive immune responses. Of the 11 human TLRs identified so far, TLR1, TLR2, TLR4, TLR5, and TLR6 are expressed on the plasma membrane, while TLR3, TLR7, TLR8, and TLR9 are localized on the endosomal and lysosomal membranes and recognize viral nucleic acids (dsRNA, ssRNA, recognized by *TLR7*) and methylated CpG motifs in DNA (recognized by TLR9). These receptors recognize various exogenous molecules of microbial origin, named pathogen-associated molecular patterns (PAMPs), as well as endogenous molecules, called damage-associated molecular patterns (DAMPs) or alarmins, which originate from host cells after tissue damage or cell death [11–13]. When distinct PAMPs or DAMPs stimulate TLRs through ligand binding, the recruitment of specific adapters and their homotypic interactions are initiated, activating the transcription factors nuclear factor kappa light chain (NFκB) and IFN regulatory factor (IRFs), as well as phosphoinositide 3-kinase/protein kinase B (PI3K/AKT) and mitogen-activated protein kinase (MAPK) cascade [11]. The binding of PAMP/DAMP to TLRs on the cell surface of immature antigen-presenting cells (APCs) leads to their activation into mature cells that can present antigens to T lymphocytes and subsequently initiate immune responses [13]. Thereby, TLRs trigger a variety of signaling cascades that activate innate and adaptive immunity and regulate the expression of inflammatory cytokines and chemokines.

In addition to the well-known role of TLRs in innate immune response, TLRs have also been linked to malignant transformation and immune surveillance of tumors [12]. TLRs are not only expressed on immune cells involved in the antitumor immune response but also show aberrant expression on cells of various tumor types, including OSCC [14, 15]. TLRs play dual roles in cancer cells and the tumor microenvironment. By recognizing DAMPs from tumor cell debris or PAMPs associated with oncogenic viruses, TLRs initiate antitumor immune responses by triggering the release of pro-inflammatory cytokines and chemokines, inducing angiogenesis, vascular permeability, and cell survival, which promote immune cell infiltration into tumors [12]. In the tumor microenvironment of various solid tumors, including HNSCC, TLRs induce the immunostimulatory potential of immune cells and the generation of tumor-reactive T cells, thereby promoting the antitumor immune response [16]. On the other hand, uncontrolled TLRs signaling leads to chronic inflammation, provides a tumor microenvironment necessary for increased cancer cell proliferation and survival, and contributes to tumor cell evasion and chemoresistance [17]. Currently, TLRs are being investigated as prognostic markers and pharmacologic targets for oncological treatment in various cancer types [13].

Recent studies have linked variations to *TLR* genes with distinct contributions to host defense, modulating individual susceptibility to various infectious, inflammatory, and malignant diseases. Evolutionary findings revealed that *TLR7* and *TLR9* are among the least diverse *TLR*s genes worldwide and that mutations affecting these intracellular sensors exert more deleterious effects compared to TLRs expressed on the cell membrane [18]. Several polymorphisms in the *TLR9* gene, including functional polymorphism −1486 C/T (rs187084), situated in the promoter of the *TLR9* gene in 3p21.3 locus, have been associated with cervical and endometrial cancer [19, 20], as well as gastric cancer [21, 22]. The *TLR7* gene polymorphism rs3853839 (C/G), located in the 3 untranslated region (UTR) on the X chromosome (Xp22.2), was previously associated with survival in colorectal carcinoma [23]. However, there are limited data on the association between *TLR7* and *TLR9* polymorphisms and prognosis or susceptibility to oral cancer [24, 25] or other subtypes of HNSCC, such as nasopharyngeal carcinoma (NPC) [26–28]. Furthermore, clinical studies are showing promising effects of *TLR9* and *TLR7* agonists in enhancing the anti-tumor activity in HNSCC [29–31]. Therefore, the main objective of this study was to examine the potential association of single nucleotide polymorphisms (SNPs) in 3′ UTR of *TLR7* gene (rs3853839) and *TLR9* −1486 T/C (rs187084) with cancer susceptibility, clinicopathological features, prognosis, and overall survival (OS) of HPV-negative OSCC patients.

## Materials and methods

### Study group and biological samples

The study group consisted of 95 patients with histologically confirmed OSCC treated at the Clinic for Maxillofacial Surgery, Military Medical Academy, Belgrade, Serbia, between 2008 and 2020. Experienced pathologists performed cancer staging based on the eighth edition of the American Joint Committee on Cancer (AJCC) TNM classification for oral and oropharyngeal tumors. Inoperable patients were excluded from this study. All OSCC patients received additional transcutaneous antitumor radiotherapy four to six weeks after surgical treatment according to the protocol for this cancer type.

Tissue samples of OSCC were collected at the time of surgery, then frozen and stored at −70 ^∘^C until DNA isolation. The control group consisted of 107 age- and sex-matched healthy controls, without previous cancer diagnosis. Since germline polymorphisms are inherited genetic variants present in all body cells, they can be detected from DNA in readily available normal tissue such as peripheral blood, eliminating the need for oral tissue samples in healthy individuals. Therefore, the collection of peripheral blood from healthy controls was conducted through venipuncture, and the samples were stored at −40 ^∘^C until DNA isolation. The participants in both OSCC cohort and control group were Caucasians of the Serbian population.

Data on alcohol consumption, smoking status, and demographics were gathered through in-person interviews. Due to the limited number of non-smokers, all patients with OSCC were divided into two groups: those who currently smoke or have ever smoked cigarettes, and those who had never smoked in the past. Both the OSCC group and healthy individuals were classified as light drinkers if they consumed 3 or less drinks per week, moderate if they consumed 4–14 drinks per week, and heavy drinkers if they consumed more than 14 alcoholic drinks per week. Both hospital data and self-reporting are used to some extent in this information. Due to incomplete hospital data or biased self-reporting data on alcohol consumption, patients were divided into two groups: non-to-low drinkers and moderate-to-heavy drinkers.

**Table 1:** Demographic characteristics in oral squamous cell carcinoma cases and controls

**Variables**		**Controls**		**OSCC**		***P***
		***n* ═ 107**	**%**	***n* ═ 95**	**%**	
Sex	Male	72	67.29	71	74.74	0.315
	Female	35	32.71	24	25.26	
Age*	<58	53	49.53	45	47.37	0.759
	≥58	54	50.47	50	52.63	
Smoking	Never	67	62.62	26	27.37	**0.0001**
	Current/former	40	37.38	69	72.63	
Alcohol consumption	None/low	89	83.18	65	68.42	**0.014**
	Moderate/high	18	16.82	30	31.58	

*Age according to median value of 58 years. Bold values represent statistical significance (*P* < 0.05). OSCC: Oral squamous cell carcinoma.

### DNA isolation, genotyping, and HPV status

According to the manufacturer’s instructions, DNA isolation from OSCC tissue samples was performed using the TRIZOL reagent (Invitrogen, Darmstadt, Germany). The PureLinkTM Genomic DNA Kit (Thermo Fisher Scientific, Waltham, MA, USA) was used to isolate DNA from the blood samples of the control group. Using the commercially available TaqMan SNP genotyping assays on Real-Time PCR 7300 (Applied Biosystems, Foster City, CA, USA), the allelic discrimination method was used to genotype the polymorphisms rs3853839 in the *TLR7* gene and rs187074 in the *TLR9* gene. Basic information regarding the SNPs and assays is provided in Table S1.

The HPV High Risk Screen Real-TM Quant kit (Sacace) was used to determine the tumor tissues’ HPV status, and the beta-globine gene was used as an internal control. The kit covers 12 different HPV types (16, 18, 31, 33, 35, 39, 45, 51, 52, 56, 58, and 59), including both high-risk (16, 18, 31, and 45) and intermediate-risk HPV types (33, 35, 39, 51, 52, 56, 58, and 59).

### Bioinformatics analysis

The potential effect of the candidate polymorphisms on enhancers and/or transcription factor binding motifs was predicted using the HaploReg v4.2 platform [32].

### Ethical statement

The Ethics Committee of the Military Medical Academy in Belgrade, Serbia, approved the study (grant MFVMA/03/23-25, approval number 6/4/2023) in accordance with the Helsinki Declaration (1964) and subsequent amendments. Informed consent was obtained from all study participants while maintaining confidentiality and privacy.

### Statistical analysis

SPSS software version 20.00 (SPSS Inc., Chicago, IL, USA) was employed in the statistical analyses. The contingency tables were assessed to compare genotype data with clinicopathological features using either the chi-square test or Fisher’s exact test. To evaluate the risk of oral cancer using a population-based case-control design, the prevalence of genetic variants was compared between OSCC patients (cases) and controls (healthy individuals). Unconditional binary logistic regression was used to determine the odds ratio (OR) and 95% confidence interval (CI) after adjusting for sex and age, as potential confounders. A log-rank test was performed to compare Kaplan–Meier survival curves between genotypes. Univariate and multivariate Cox proportional hazard regression analysis was assessed to estimate the hazard of an event, death as the primary endpoint for OS, or recurrence for recurrence-free survival (RFS), based on hazard ratio (HR) and its 95% CI. The HR for a single predictor variable was determined using univariate Cox regression. Next, the statistically significant variables from the univariate analysis and those with a significance level below 20% were analyzed simultaneously using the multivariate Cox regression model to examine the relationship between multiple, potentially interacting risk factors and patient survival. The multivariate Cox regression was run using the backward stepwise method, which eliminated variables with *P* < 0.1 from the model. In the final multivariable Cox model, only variables significantly associated with survival remain as independent risk factors for worse OS or RFS.

## Results

Demographic characteristics of both OSCC patients and controls, matched by age and sex, are shown in Table 1. Smoking and high alcohol consumption were more common in the OSCC group (*P* ═ 0.0001, and *P* ═ 0.014, respectively) (Table 1). The OSCC patient cohort consisted of 71 men and 24 women. The patients’ ages ranged from 39 to 80 years, with an average age of 58. Stage II tumors had 25.3% of patients (24 out of 95), while stage III tumors had 74.7% of patients (71/95) (Table 2). The mean follow-up time was 34.5 months, and the median was 30 months (range 9–100 months). The recurrence rate was 58.9% (56/95 patients). The recurrence period ranged from 1 to 42 months, with an average recurrence time of 6.3 months and a median time of 5 months. All OSCC tissue samples were HPV-negative in both high- and intermediate-risk HPV panels.

**Table 2:** Association of *TLR7* and *TLR9* gene polymorphisms with clinicopathological variables in oral squamous cell carcinoma patients

**Variables**		**N**	***TLR9* rs187084**			P/P*	TLR7 rs3853839^**^		P/P*
			**TT**	**TC**	**CC**		**CC/C-**	**G-allele carriers**	
Sex	Male	71	17	40	14	0.395/0.305	54	17	0.611
	Female	24	9	12	3		17	7	
Age (median)	<58	45	12	24	9	0.879/0.920	34	11	0.862
	≥58	50	14	28	8		37	13	
Smoking	Never	26	9	13	4	0.619/0.475	20	6	0.763
	Current/former	69	17	39	13		51	18	
Alcohol consumption	Non/low	65	20	38	7	**0.027/**0.396	42	23	**0.001**
	Moderate/high	30	6	14	10		29	1	
Histological grade	1	30	5	18	7	0.249/0.180	25	5	0.190
	2/3	65	21	34	10		46	19	
Nuclear grade	1	17	2	13	2	0.131/0.196	12	5	0.664
	2/3	78	24	39	15		59	19	
Nodal metastases	Absent	25	12	11	2	**0.020/0.015**	17	7	0.611
	Present	70	14	41	15		54	17	
Tumor size	T1/2	71	22	37	12	0.396/0.438	53	18	0.973
	T3/4	24	4	15	5		18	6	
Stage	II	24	11	11	2	**0.047/0.037**	17	7	0.611
	III	71	15	41	15		54	17	
Recurrence	Absent	39	12	23	4	0.265/0.105	28	11	0.636
	Present	56	14	29	13		43	13	

*P values for combined wild-type (WT) and heterozygote (HT) genotypes vs mutated (MT) homozygote genotype, ^**^For *TLR7* rs3853839, located on X chromosome, CC/C- represents CC genotype in females, or C allele in male patients; G-allele carriers represent heterozygote and mutated homozygote genotypes (CG+GG) in females or genotype G- in male patients. Bold values represent statistical significance (*P* < 0.05). TLR: Toll-like receptor.

### *TLR9* and *TLR7* polymorphisms association with clinicopathological features

The relationship between the polymorphisms in the *TLR7* and *TLR9* genes and the clinicopathological features of the OSCC cohort is summarized in Table 2. The presence of lymph node metastases was associated with a higher prevalence of CC variant genotype of *TLR9* rs187084 polymorphism compared to the major TT genotype (*P* ═ 0.020) and compared to T-allele carriers (combined TT + CT), *P* ═ 0.015. Additionally, a greater frequency of advanced stage III was seen in patients with the variant CC genotype of the *TLR9* rs187084 polymorphism compared to patients with major TT genotype (*P* ═ 0.047) and compared to T-allele carriers (combined TT + CT genotypes), *P* ═ 0.037. The *TLR7* polymorphism rs3853839 was not associated to any of the clinicopathological features. A higher prevalence of the *TLR9* rs187084 variant CC genotype (*P* ═ 0.027), but a lower prevalence of *TLR7* rs3853839 variant G-allele carriers (*P* ═ 0.001) was observed in heavy drinking patients (Table 2).

### Kaplan–Meier and Cox regression analysis

**Figure 1.**
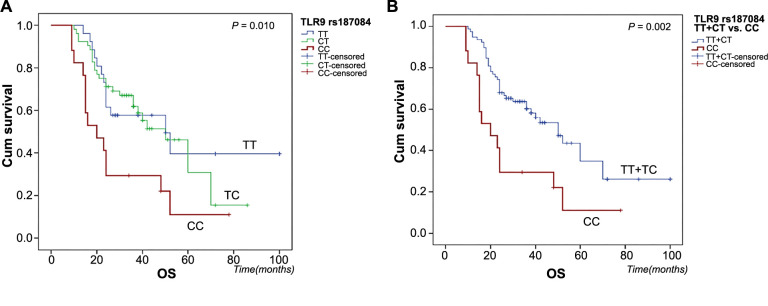
**Kaplan–Meier curves of *TLR9* rs187084 genotypes association with OS of OSCC patients, compared by log-rank test.** *P* values were calculated according to the log-rank test. OS curves for (A) TT, CT, and CC genotypes of *TLR9* rs187084 polymorphism; (B) combined TT and CT genotypes vs variant CC genotype. OS: Overall survival; OSCC: Oral squamous cell carcinoma; Cum: Cumulative.

Kaplan–Meier analysis revealed that patients with the mutated CC genotype of the *TLR9* rs187084 polymorphism had a lower OS rate (*P* ═ 0.010, log-rank test), as shown in Figure 1A. When comparing the CC variant of the *TLR9* rs187084 polymorphism to T-allele carriers (combined TT + CT genotypes), the OS rate was even lower (*P* ═ 0.002), as Figure 1B illustrates. *TLR9* rs187084 polymorphism did not predict the RFS of OSCC patients (*P* ═ 0.359). The *TLR7* polymorphism rs3853839 had no impact on OS or RFS in our cohort (*P* ═ 0.832 and *P* ═ 0.739, respectively).

In our HPV-negative OSCC cohort, smoking, alcohol use, stage, nodal status, tumor size, recurrence, and *TLR9* rs187084 polymorphism were significant prognostic factors for OS, according to univariate Cox regression analysis (Table 3). To explore the independent predictors, Cox proportional hazard regression was used for multivariate survival analyses. Among the variables included in the multivariate analysis were those that were statistically significant in the univariate analysis and those with a significance level of less than 10%. Nodal status and recurrence remained an independent predictor of OS in the multivariate model (*P* ═ 0.001 and *P* ═ 0.0001, respectively), while *TLR9* polymorphism rs187084 lost its significant association with mortality in the final model.

**Table 3:** Univariate analysis of different prognostic factors in relation to recurrence-free survival and overall survival, according to Cox proportional hazards regression analysis

**Cox analysis models**		**Overall survival**		**Recurrence-free survival**	
		**HR** **(95% CI)**	***P***	**HR** **(95% CI)**	***P***
Univariate	Sex	0.532 (0.260–1.092)	0.085	1.278 (0.869–1.879)	0.212
	Age	1.665 (0.959–2.892)	0.070	1.103 (0.767–1.584)	0.597
	Smoking	2.220 (1.085–4.544)	**0.029**	1.169 (0.600–2.277)	0.645
	Alcohol consumption	2.195 (1.325–3.636)	**0.002**	1.118 (0.705–1.774)	0.635
	Nuclear grade	1.283 (0.878–1.873)	0.198	0.790 (0.514–1.194)	0.284
	Histological grade	0.962 (0.727–1.272)	0.785	0.912 (0.697–1.194)	0.502
	Stage	6.821 (2.338–15.374)	**0.0003**	2.074 (1.028–4.186)	**0.042**
	Nodal status	6.139 (2.214–17.026)	**0.0005**	2.082 (1.035–4.189)	**0.040**
	Tumor size	1.662 (1.209–2.175)	**0.001**	1.090 (0.809–1.468)	0.572
	Recurrence	3.899 (2.065–7.361)	**0.00003**	–	–
	*TLR9*	1.638 (1.076–2.492)	**0.021**	1.278 (0.869–1.879)	0.212
	*TLR7*	0.839 (0.682–1.365)	0.774	1.103 (0.767–1.584)	0.597
Multivariate^#^	Nodal status	5.396 (1.939–15.016)	**0.001**	2.082 (1.035–4.189)	**0.040**
	Recurrence	3.492 (1.844–6.614)	**0.0001**	–	–

**^#^**Variables that significantly associate with survival remained in the final multivariate Cox regression model and are presented in the table, while marginally significant variables (0.05 < *P* < 0.1) remained in the final multivariate model, but are not presented. Bold values represent statistical significance (*P* < 0.05). HR: Hazard ratio; CI: Confidence interval; TLR: Toll-like receptor.

### *TLR9* and *TLR7* polymorphisms and OSCC risk

A population-based case-control section of our study examined the association of *TLR7* and *TLR9* genetic variants with susceptibility to oral cancer, by comparing the frequencies of the *TLR7* and *TLR9* genetic variants between patients with OSCC (cases) and healthy individuals, without a prior history of cancer (controls). The distribution of *TLR9* rs187084 (T/C) and *TLR7* rs3853839 (C/G) gene polymorphisms in OSCC patients compared to the control group is outlined in Table 4. There was no association found between the analyzed genetic polymorphisms and OSCC susceptibility.

**Table 4:** Association between *TLR7* and *TLR9* gene polymorphisms and OSCC risk

***TLR* polymorphism**	**Genotype**	**Controls, *n* ═ 107** **(%)**	**OSCC, *n* ═ 95 (%)**	**Adjusted OR (95% CI)**	***P***
*TLR9* rs187084 (T/C)	TT	36 (33.64)	26 (27.37)	1.237 (0.811–1.886)	0.324
	TC	56 (52.34)	52 (54.74)		
	CC	15 (14.02)	17 (17.89)		
*TLR7* rs3853839^**^ (C/G)	CC/C-	89 (83.18)	65 (68.42)	1.303 (0.921–1.845)	0.135
	G-allele carriers	18 (16.82)	30 (31.58)		

**For *TLR7* rs3853839, located on X chromosome, CC/C- represents CC genotype in females, or C allele in male patients; G-allele carriers represents heterozygote and mutated homozygote genotypes (CG+GG) in females or genotype G- in male patients. OR: Odds ratio; CI: Confidence interval; OSCC: Oral squamous cell carcinoma; TLR: Toll-like receptor.

### Bioinformatics analysis

Enhancer histone marks and motif changes caused by investigated polymorphisms can result in allele-specific binding of transcription factors, according to our analysis of HaploReg v4.1, a platform for chromatin states and regulatory motif changes. The *TLR9* rs187084 polymorphism leads to significant chromatin modifications, especially in primary T cells, naive T helper cells and memory cells, as well as regulatory T cells, which could influence the motif for possible binding of LUN-1, Maf, and Nkx2. On the other hand, *TLR7* rs353547 causes less significant motif changes in potential binding sites of NF-AT, Ik-2, HDAC2, and EWSR1-FLI1 (Table S1).

## Discussion

Despite remarkable efforts in the development of new diagnostic and therapeutic modalities, oral carcinomas still have a high recurrence rate, a low five-year survival rate, and an increasing prevalence [1]. Given that immune pathways impact cancer susceptibility, progression, survival, and therapy, there is growing interest in the pharmacological targeting of *TLR*s and the role of their genetic polymorphisms in cancer. However, there are limited data on *TLR7* and *TLR9* polymorphisms associated with OSCC [25] and HNSCC [26–28].

According to our findings, the *TLR9* rs187084 polymorphism could be related to the prognosis of patients with HPV-negative OSCC. Our results suggest that OSCC patients with the variant CC genotype or C allele carriers (CT+CC genotypes) of the *TLR9* rs187084 polymorphism have a higher risk of lymph node metastasis and advanced tumor stage, as well as a reduced OS, compared to patients with the TT genotype. However, logistic regression analysis showed that *TLR9* rs187084 is not an independent prognostic factor in our HPV-negative OSCC cohort. We also found a significant correlation between OSCC patients’ high alcohol consumption and *TLR7* and *TLR9* polymorphisms. Both the OSCC cohort and the control group had similar *TLR9* and *TLR7* genotype distributions, indicating that these genetic variations are not associated with OSCC susceptibility.

To the best of our knowledge, our study provides the first report that the functional polymorphism rs187084 (−1486T/C) in the *TLR9* gene may be associated with progression and survival in oral cancer patients. Consistent with our findings, the studied *TLR9* rs187084 polymorphism was previously associated with advanced tumor stage and lymph node metastasis in NPC, another subgroup of HNSCC [26]. Another study associated different *TLR9* polymorphism rs5743836 (−1237T/C), but not rs187084, with NPC progression and survival [28].

The functional relevance of the *TLR9* rs187084 (−1486T/C) polymorphism has been previously shown in pulmonary tuberculosis, demonstrating that the variant C allele of this polymorphism increases the transcriptional activity of *TLR9*, which in turn reduces the expression of inflammatory cytokines such as IFNγ and TNFα [33]. Furthermore, patients with the variant genotype CC of the *TLR9* rs187084 polymorphism had significantly higher VEGF protein expression in NPC than patients with the major genotype TT [26]. These findings indicate that pro-tumor effects of *TLR9* are also mediated independently of NF-κB activation, through the induction of other regulatory proteins involved in carcinogenesis. According to the previous bioinformatics analysis, the rs187084 polymorphisms affect the *TLR9* gene’s binding sites for multiple transcription factors, including RELA, NFKB1, and THAP1 [34]. Previous in silico analysis indicated that *TLR9* rs187084 polymorphism creates a putative Sp1 binding site, which could modify transcriptional activity and the *TLR9* gene expression [35]. Furthermore, our HaploReg v4.1 analysis, a platform for studying chromatin states and regulatory motif changes, revealed that the *TLR9* rs187084 polymorphism causes significant chromatin modifications, especially in T lymphocyte subsets, naive T helper and memory cells, regulatory T cells, CD8+ T cells, and natural killer cells. This polymorphism may influence the motif for potential binding of transcription factor LUN-1, MAF oncogene family, and a cancer stem cell regulator NK2 homeobox 1 (Nkx2), previously associated with oral cancer in vitro [36].

The functional relevance of *TLR7* gene polymorphism rs3853839 (C/G) was previously demonstrated in systemic lupus erythematosus (SLE), where G-allele carriers had increased *TLR7* mRNA expression and more enhanced IFN production than C-allele carriers [37]. However, our HaploReg v4.1 analysis showed that *TLR7* rs353547 leads to less noticeable motif changes at the NF-AT, Ik-2, HDAC2, and EWSR1-FLI1 binding sites, mainly on monocytes, neutrophils, and B cells, which may be consistent with our results showing that this polymorphism has less noticeable effects on antitumor immune response in OSCC.

Our results suggesting that high-expressing CC genotype of *TLR9* rs187084 polymorphism is associated with heavy alcohol drinking in OSCC patients are in line with previous findings demonstrating that heavy alcohol consuming associated with higher levels of *TLR9*, and has an impact on particular immunophenotypes and poor survival of OSCC patients [38]. Furthermore, our results show that *TLR7* high-expressing G-allele carriers are less frequent in high alcohol-drinking HPV-negative OSCC patients. Alcohol abuse could cause damage to oral mucosa cells and the release of DAMPs, which trigger TLR signaling that induces the expression of various genes, including NFκB, a key regulator of the immune response and inflammation. *TLR9* and *TLR7* polymorphisms could potentially have a role in the modulation of immune responses to alcohol-associated DAMPs, and the impaired initiation of anti-tumor immune response in oral cancer.

While our study did not show the association of *TLR7* and *TLR9* polymorphisms with OSCC risk, several studies on oral cancer and other tumor types demonstrated their association with cancer susceptibility. A study on *TLR9* polymorphisms rs5743836, rs352140, rs187084, and rs352139 in OSSC patients found that all investigated variants could increase the oral cancer risk and potentiate bacterial gingival inflammation/gingival recession [25]. Accordingly, TC and CC genotypes and the C allele of *TLR9* rs187084, as well as *TLR9* haplotypes containing *TLR9* rs187084 C allele were associated with increased OSCC risk, while haplotypes containing the T allele may play a protective role [25]. A large meta-analysis study showed that the investigated *TLR9* polymorphism rs187084 was significantly associated with an increased risk of overall cancer, particularly cervical cancer, whereas the different polymorphisms rs352140 and rs5743836 in the *TLR9* gene exerted a protective role in the development of breast and digestive cancers [39]. More recent meta-analyses showed that *TLR9* rs187084 polymorphism, among others, is significantly associated with increased susceptibility to cervical cancer [34, 40]. Moura et al. [34] suggested that polymorphisms in the *TLR9* gene could affect intracellular signaling and alter host immune response patterns, leading to an increased risk of cervical cancer. Furthermore, patients with CC+CT genotypes of rs187084 and CT+TT genotypes of rs352140 *TLR9* gene polymorphisms had multiple and persistent high-risk HPV cervical infections, as well as longer time to clearance [41], suggesting an essential role of *TLR9* in antiviral immune response. Since oral cancer is closely associated with HPV infection, *TLR7* and *TLR9* as endogenous sensors of viral nucleic acids may play a significant role in the malignant transformation in OSCC [15]. However, the expression of *TLR7* and *TLR9* varies between HPV-positive and HPV-negative OSCC. While *TLR9* expression is higher in more aggressive HPV-negative tumors, *TLR7* expression is higher in HPV-positive tumors [42, 43], which suggests *TLR9* and *TLR7* may have opposing effects and both play roles in OSCC that go well beyond virus recognition.

Our findings regarding the correlation between advanced stage and poor survival of OSCC patients with the high-expressing variant allele C of *TLR9* polymorphism may be in line with previous findings associating the high *TLR9* expression with prognosis and survival in multiple carcinomas. *TLR9* is upregulated in human OSCC in vitro and in vivo, and its high expression correlates with advanced tumor stage [44, 45] and decreased survival rates [38]. The expression of *TLR9* significantly increased from oral dysplasia to OSCC, reflecting the role of *TLR9* in progression during OSCC malignant transformation [46]. A meta-analysis revealed that overexpression of *TLR9* or *TLR1-5* negatively correlated with clinical outcomes in patients with HNSCC [47]. A recent study suggests that poor outcomes in head and neck cancer may be associated with high expression of *TLR9* and *p53* [48]. Thus, it seems that the activation of *TLR9* is closely linked to oral cancer prognosis, which implies that the inhibition of *TLR9* seems to be a targeting point of future investigation [49]. Furthermore, high *TLR9* expression has previously been associated with a reduced OS rate in glioblastoma [50] and hepatocellular carcinoma [51]. *TLR9* signaling has also been found to promote tumor progression in lung cancer [52] and colorectal cancer [53], and accelerate cell invasion in prostate [54], ovarian [55], and breast cancer [56]. Overexpression of *TLR9* was associated with the induction of epitheliomesenchymal transition (EMT) and deregulation of EGFR signaling in invasive breast carcinomas [57].

Recent studies revealed variable data regarding *TLR7* tumor suppressing role in various cancer types, including OSCC [58]. Polymorphisms in *TLR7* and *TLR5* were recently associated with oral cancer suppression, while polymorphisms in *TLR4* and *TLR2* were associated with oral cancer progression [24]. However, our results did not associate *TLR7* polymorphism with clinical features, survival, or oral cancer susceptibility, in line with previous findings in lung [59], or gastric cancer [60].

The dual role of *TLR9* and *TLR7* in cancer is complex and their effects can be context-dependent, depending on various factors, including the specific type of cancer and tumor stage. *TLR9* has been shown to function as a sensor for DNA released from tumors following chemotherapy [61]. Tumor-derived DNA stimulates dendritic cells in a *TLR9*-dependent manner, causing them to proliferate, take up antigens, mature within the tumor, and subsequently migrate to the draining lymph nodes to prime tumor-specific CTLs [61], indicating an essential role of *TLR9* in anti-tumor immunity. Activated *TLR9*, by CpG DNA in infection-induced cancers, or by tumor-derived DAMPs, induces NF-κB activation, resulting in immune cell infiltration, increased proliferation, and reduced apoptosis, as has been demonstrated in human prostate cancer [62]. Through activator protein-1 (AP-1)-mediated control of cyclin D1 expression, *TLR9* contributes to the proliferation of OSCC cells [63]. Furthermore, *TLR9* activation induces tumorigenesis and immune escape via PD-L1 upregulation in OSCC [44] and hepatocellular carcinoma [64]. Suppression of *TLR9* decreases PD-L1 expression and inhibits immune evasion, invasion, migration, and proliferation through inhibition of poly(ADP-ribose) polymerase (PARP1) [44].

In the pancreatic cancer model, *TLR7* signaling through STAT3 results in loss of PTEN, p16, and cyclin D1, which leads to tumor development [65]. By upregulating pro-inflammatory cytokines, anti-apoptotic protein Bcl-2, and angiogenesis-related VEGFR2, *TLR7* activation in lung cancer increases tumor cell survival and resistance to apoptosis [66]. In NSCLC, *TLR7* signaling contributes to immune evasion by suppressing the activity of CTLs by recruiting immunosuppressive MDSCs to the tumor microenvironment, facilitating tumor progression and metastasis [67].

The anti-tumor immune response is regulated by the interplay between agonist signals, enhancing the anti-tumor immunity and antagonists that block immune signaling. *TLR9* agonists hold great potential in cancer therapy, showing the synergistically enhanced immunostimulatory capacity when used in combination with other agents, chemotherapy, radiation, or checkpoint inhibitors. Despite the fact that *TLR9* agonists alone were unable to elicit significant systemic antitumor immune responses due to their immunosuppressive effects when combined with an anti-PD-1 antibody or anti-PD-L1, they yielded an additive effect via negative regulation of PARP1/STAT3 in a clinical trial in hepatocellular carcinoma [64]. An ongoing multicenter, phase 2 study (NCT04633278) of intratumoral administration of CMP-001, a virus-like particle containing CpG-based *TLR9* agonist, in combination with intravenous pembrolizumab is currently being evaluated in patients with recurrent or metastatic HNSCC [30]. This *TLR9* agonist has previously successfully induced an antitumor immune response via the production of IFN-α by plasmacytoid dendritic cells (pDCs), resulting in increased expression of PDL1, IDO, and CD80 and suppressed expression of CXCL10, but also led to increased proliferation of autologous CD4 T cells [68]. In situ vaccination of CMP-001 TLR-9 agonist in combination with anti-PD-1 therapy induced durable HNSCC tumor regression at injected tumors and distant ones and significantly prolonged survival compared with anti-PD-1 therapy alone [29], indicating the higher effectiveness of local administration compared to systemic administration. However, *TLR9* agonists’ immunostimulatory effects may be influenced by host *TLR9* polymorphisms. Using a tailored strategy based on the patient’s *TLR9* genotype could potentially make *TLR9* agonist therapy in cancer more effective in the future.

Several limitations in this study should be acknowledged. First, a limited number of patients and only two *TLR* polymorphisms were included in this study. Second, both hospital data and self-reporting are used to some extent for alcohol consumption information, which may lead to incorrect estimates due to impression management bias. Finally, the results were obtained in the individual Caucasian–Serbian population and our results may not generalize to other Caucasian groups or the general population.

## Conclusion

Our findings indicate that *TLR9* rs187084 could be a significant prognostic biomarker of worse prognosis and demonstrate its association with lymph node metastases, advanced tumor stage, and poor survival of HPV-negative OSCC patients. Our results may indicate the potential clinical utility of *TLR9*. Through altered sensoring capacity and NF-κB activation, polymorphisms in the *TLR9* gene may impact immune cell recruitment and the antitumor immune response. Polymorphisms in immune-related genes can affect the overall immune background and potentially disrupt the dynamic equilibrium of the tumor microenvironment. Given the limitations of the present study, independent, large-scale, prospective studies in diverse populations are needed to further validate our results and fully elucidate the underlying biological mechanisms of *TLR9* and *TLR7*.

## Supplemental data

**Table S1:** Characteristics of genotyped *TLR7* and *TLR9* polymorphisms and HaploReg v4.2 prediction results

**Gene**	**rs number**	**Location**	**SNP change**	**Region**	**HaploReg v4.2 prediction**		
					**Enhancer histone marks**	**Cell types**	**Motifs changed**
*TLR7*	rs3853839	Xp22.2	C/G	3′ UTR	+	Neutrophils, monocytes, B cells	NF-AT, Ik-2, HDAC2, EWSR1-FLI1
*TLR9*	rs187084	3p21.2	T/C	Promoter	+++	Primarily T cell subsets	LUN-1, Maf, Nkx2

SNP: Single nucleotide polymorphism; NF-AT: Nuclear factor of activated T-cells; HDAC2: Histone deacetylase 2; TLR: Toll-like receptor.

## Data Availability

For privacy and/or ethical reasons, clinical data from human participants are not publicly available unless specifically requested by contacting the corresponding author.
